# Automated pipeline for nerve fiber selection and g-ratio calculation in optical microscopy: exploring staining protocol variations

**DOI:** 10.3389/fnana.2023.1260186

**Published:** 2023-11-22

**Authors:** Bart R. Thomson, Louise Françoise Martin, Paul L. Schmidle, Hannah Schlierbach, Anne Schänzer, Henning Richter

**Affiliations:** ^1^Department of Neurosurgery, Clinical Neuroscience Center, University Hospital and University of Zurich, Zurich, Switzerland; ^2^Institute of Laboratory Animal Science, Vetsuisse Faculty, University of Zurich, Zurich, Switzerland; ^3^Department of Dermatology, University Hospital Muenster, Muenster, Germany; ^4^Institute of Neuropathology, Justus Liebig University Giessen, Giessen, Germany; ^5^Diagnostic Imaging Research Unit (DIRU), Clinic for Diagnostic Imaging, Vetsuisse Faculty, University of Zurich, Zurich, Switzerland

**Keywords:** g-ratio, sciatic nerve, mice, optical microscopy, deep learning, segmentation

## Abstract

G-ratio is crucial for understanding the nervous system’s health and function as it measures the relative myelin thickness around an axon. However, manual measurement is biased and variable, emphasizing the need for an automated and standardized technique. Although deep learning holds promise, current implementations lack clinical relevance and generalizability. This study aimed to develop an automated pipeline for selecting nerve fibers and calculating relevant g-ratio using quality parameters in optical microscopy. Histological sections from the sciatic nerves of 16 female mice were prepared and stained with either p-phenylenediamine (PPD) or toluidine blue (TB). A custom UNet model was trained on a mix of both types of staining to segment the sections based on 7,694 manually delineated nerve fibers. Post-processing excluded non-relevant nerves. Axon diameter, myelin thickness, and g-ratio were computed from the segmentation results and its reliability was assessed using the intraclass correlation coefficient (ICC). Validation was performed on adjacent cuts of the same nerve. Then, morphometrical analyses of both staining techniques were performed. High agreement with the ground truth was shown by the model, with dice scores of 0.86 (axon) and 0.80 (myelin) and pixel-wise accuracy of 0.98 (axon) and 0.94 (myelin). Good inter-device reliability was observed with ICC at 0.87 (g-ratio) and 0.83 (myelin thickness), and an excellent ICC of 0.99 for axon diameter. Although axon diameter significantly differed from the ground truth (*p* = 0.006), g-ratio (*p* = 0.098) and myelin thickness (*p* = 0.877) showed no significant differences. No statistical differences in morphological parameters (g-ratio, myelin thickness, and axon diameter) were found in adjacent cuts of the same nerve (ANOVA *p*-values: 0.34, 0.34, and 0.39, respectively). Comparing all animals, staining techniques yielded significant differences in mean g-ratio (PPD: 0.48 ± 0.04, TB: 0.50 ± 0.04), myelin thickness (PPD: 0.83 ± 0.28 μm, TB: 0.60 ± 0.20 μm), and axon diameter (PPD: 1.80 ± 0.63 μm, TB: 1.78 ± 0.63 μm). The proposed pipeline automatically selects relevant nerve fibers for g-ratio calculation in optical microscopy. This provides a reliable measurement method and serves as a potential pre-selection approach for large datasets in the context of healthy tissue. It remains to be demonstrated whether this method is applicable to measure g-ratio related with neurological disorders by comparing healthy and pathological tissue. Additionally, our findings emphasize the need for careful interpretation of inter-staining morphological parameters.

## 1 Introduction

G-ratio is a quantitative measure of relative myelin thickness around an axon, which is given by the ratio of the inner and outer diameter of the myelin sheath. Both the myelin thickness and axon diameter contribute to the neuronal conduction velocity, and its ratio is important for understanding the health and function of the nervous system. Abnormalities in myelination can occur in a variety of neurological disorders, including multiple sclerosis, traumatic brain injury, and neurodegenerative diseases (Fields, 2008). Moreover, it has been proposed that the g-ratio, steered by testosterone differences, is dependent on gender during development (Paus and Toro, 2009; Perrin et al., 2009; Pesaresi et al., 2015). Measuring the g-ratio can provide valuable insights into developmental mechanisms, the pathology of disorders, as well as aid in diagnosis and treatment. This research study aims to enhance the analysis of the g-ratio in healthy animals by mitigating potential confounding factors that should be addressed prior to evaluating pathological conditions.

Manual g-ratio measurement is a time-consuming and labor-intensive process, prone to observer bias and variability. An automated and standardized technique for g-ratio calculation can benefit the field of neuroscience by providing a more reliable, efficient, and objective method for quantifying myelination. Thereby, reproducibility and accuracy of results are expected to increase. Early advances in axon and myelin segmentation, and morphological analysis rely on traditional image processing techniques (More et al., 2011; Liu et al., 2012; Bégin et al., 2014; Zaimi et al., 2016; Kaiser et al., 2021). These approaches are designed for a specific image processing task, and struggle with different contrast or morphology than what they were designed for. Additionally, image specific preprocessing often limits inference on unseen novel data (Öztürk and Akdemir, 2018).

Algorithms based on deep learning overcome these issues since they are context aware, and often scale and rotation invariant, in part due to data augmentation (Marcos et al., 2016). Current implementations (Table 1) only segment axons (Deng et al., 2021) or often rely on small input patches (Naito et al., 2017) and are not designed to work with background noise that is present in whole slide histological sections. Implementations focusing on axon counting have been proposed but do not provide value regarding the quality of the performed staining (Reynaud et al., 2012; Zarei et al., 2016; Goyal et al., 2023a,b). Currently, the best performing publicly available algorithm is trained on EM histological sections (Zaimi et al., 2018), for which others have shown strong generalizability to optical microscopy (OM) with (Daeschler et al., 2022) and without (Wong et al., 2021) transfer learning. The clinical utility of these algorithmic measurements is questionable because they do not differentiate between individual nerve characteristics and those that are representative of all nerves in the specific histological section.

**Table 1:** Overview of prior nerve segmentation implementations.

Reference	Modality	Main characteristic / advantages	Clinical limitations
Zaimi et al. (2016)	OM/EM/CARS	Traditional image processing-based segmentation	Limited by user defined parameters
Zaimi et al. (2018)	EM	Fully automatic segmentation	Implementation designed for EM
Deng et al. (2021)	OM	(Un)supervised segmentation	No quality stratification
Kaiser et al. (2021)	EM	Semi-automatic GUI without code requirement	Manual corrections needed
Daeschler et al. (2022)	OM	Additional OM model to Zaimi et al. (2018)	No quality stratification
Goyal et al. (2023a,b)	OM	Morphological analysis	No quality stratification
Our implementation	OM	Nerve auto-selection for clinical analysis	Healthy-nerve-based model

Additionally, assessing g-ratio measurements obtained from diverse sources and staining techniques presents significant hurdles. Staining protocols influence the way that morphological parameters are acquired and may affect the obtained results (Ohnishi et al., 1974). Staining techniques impact the visualization and quantification of myelin, resulting in varying g-ratio values within the same animal or slide (Ward et al., 2008). This phenomenon has the potential to impact the analysis of g-ratio in histological samples and introduce intra-individual, or within-slide differences. Additionally, morphological parameters can be influenced by the species, age, health status, and sampling location of the animal from which the sample was taken (Geuna et al., 2001). These issues hamper the dependable comparison and interpretation of g-ratio across studies, potentially restricting the applicability of findings.

The primary goal of this research was to create an automated pipeline for the precise selection of nerve fibers in OM, aiming to calculate the g-ratio with clinical relevance. Furthermore, this study explores potential implications resulting from variations in staining protocols, utilizing a standardized analysis approach that we developed.

## 2 Materials and methods

### 2.1 Origin of samples and histological preparation

16 female mice (RjOrl:SWISS, Janvier; Elevage Le Genest, France) originating from another independent and unrelated study, were used for sampling of sciatic nerves. All mice were housed in a conventional facility and sampled at an age between 24 and 31 days. The healthy animals belonged to a control group and received a single injection of saline (NaCl 0.9%, 2 mL/kg, intravenous) into the lateral tail vein. The study was officially approved by the Cantonal Veterinary Office (animal permission number: ZH029/19) and follows the ARRIVE guidelines.

Euthanasia was performed according to the AVMA Guidelines for the Euthanasia of Animals by carbon dioxide inhalation at 4 weeks after injection (Underwood and Anthony, 2020). Absence of heartbeat and respiration confirmed the death of the animals, prior to sciatic nerve tissue sampling.

### 2.2 Epoxy resin embedding, staining techniques and scanning

Resin embedded sections of nerve biopsies are recommended for analysis of detailed nerve pathology (Weis et al., 2012).

All samples were fixed in 2.5% buffered glutaraldehyde and transferred to Sorensen’s phosphate buffer (pH7.2; MORPHISTO). The samples were washed in 0.1 M PBS (pH 7.2) and were processed with a tissue processor (Leica EM TP) with 1% osmium tetroxide (OsO4). Dehydration steps were followed by increasing ethanol concentrations (25, 35, 50, 70, 75, 85,100%). Prior to embedding, samples were combined with resin (Agar 100 Resin Kit; agar scientific) and were dehydrated in a desiccator overnight. Polymerization of the resin was accomplished at 60°C for 24 h. From resin blocks semi-thin cross sections (990 nm) were cut, mounted on glass slides, and stained with either p-Phenylenediamine (PPD) or Toluidine Blue (TB). Both PPD and TB are helpful for detailed imaging of peripheral nerve morphology (Weis et al., 2021). Applying PPD staining to osmicated tissue samples helps identify the lipids in the myelin sheath of peripheral nerves (Shirai et al., 2016). For PPD staining, the slides were immersed in 2% PPD (Sigma) in 100% Ethanol for 55 min at room temperature, followed by rinsing in 2 × 100% ethanol for 5 min. TB staining is a useful method to assess the number of axons and myelination (Ghnenis et al., 2018). TB staining was performed with a solution composed of 1% TB (Merck and Cie), 1% Sodium Tetraborate, and 1% Pyronin mixed at a ratio of 40:40:10. Following staining, the slides were rinsed with demineralized water. Slides were dried and cover-slipped with mounting media (Cytoseal XYL; Thermo Fisher).

All sections were scanned using NanoZoomer S360 MD (Hamamatsu) and viewed using NDP.view2 (U12388-01, Ver 2.9 Rev.2).

### 2.3 Automated segmentation of OM sections

The convolutional neural network UNet, is considered state-of-the-art in biomedical image segmentation; it contains an encoder and decoder path with skip connections to preserve low-level spatial features (Ronneberger et al., 2015). In this study, the algorithm was implemented using the MONAI framework. This framework is developed for deep learning in healthcare imaging and is freely available and open source (Cardoso et al., 2022). To obtain a ground truth for training of our algorithm, delineations were performed by 4 experienced observers using the image processing software QuPath (version 0.4.0).^1^ In the delineating process, the boundaries of each axon and its surrounding myelin sheath were used to obtain the ground truth. To guarantee standardization, all annotations were reviewed by the expert of the observers. In total, 8 nerve fibers of 4 mice containing 7,694 nerve fibers were delineated in the histological sections acquired by OM. These nerve fibers were extracted from the histological sections in 23 representative patches of 4096×4096 pixels, with a size of 0.05 μm per pixel to guarantee standardization.

The algorithm had 6 layers with 16, 32, 64, 128, 256, and 512 filters respectively, where each depth has two convolution layers followed by batch normalization and parametric rectified linear activation. In all dimensions a stride of 2 was maintained. To enhance the model’s ability to generalize across varying conditions, 512×512 voxel input patches were subject to various data augmentation (randomly rotated, flipped, and zoomed along both axes). Additionally, random Gaussian noise and smoothing filters were applied to simulate real-world variations. The model was trained using a learning rate of le-3 with Dice loss and Adam optimizer. Following the initial segmentation, cavities larger than 5 pixels were filled following the thresholding of the output probability maps at threshold 0.8.

For the model’s training and validation, the dataset was divided into 10 patches in the training set and 8 in the validation set, each containing varying axon content ranging between 17 and 1,040 axons. This stratification helped to ensure a robust training regime that could handle a wide range of axon densities. Finally, the models’ performance was evaluated by the Dice performance metric on an independent test set of 5 patches, giving an indication how well the trained model would generalize to new, unseen data. The network was trained on a local system (NVIDIA Tesla T4 GPU).

### 2.4 Training performance evaluation

During training, the algorithm’s performance was evaluated using the Dice score, defined as: Dice = (2 * (A ∩ B)) / (|A| + |B|), where A and B are the predicted and ground truth binary masks, respectively (Milletari et al., 2016).

### 2.5 Segmentation morphometrics

The patches that have been used in training and validation of the network were excluded from the morphometrical analyses in this study. Additionally, the analysis included multiple his sections of all mice in both the PPD and TB staining. Following the segmentation of the axons and myelin sheaths, post-processing is performed to exclude nerves that are not relevant, based on the morphometrics of the axon. Nerve fibers were excluded from the image if they met any of the following criteria: the axon’s shape was highly elongated (an eccentricity greater than 0.95), the axon’s structure was not compact (a solidity less than 0.9), or the axon was too small (area smaller than 50 pixels). Based on the (automated) delineation of the axon and myelin sheath, the axon diameter and myelin thickness were computed. Myelin volume fraction (MVF; ratio between area of myelin and total area of the region) and axon volume fraction (AVF; ratio between area of axon and total area of the region) were used to calculate the g-ratio (ratio between axon diameter) and myelinated fiber (axon + myelin). The g-ratio was estimated with the following formula [Stikov et al., 2015; √(1/(1 + MVF/AVF))].

Additionally, to determine the reliability of the g-ratio calculation, the extent to which measurements can be replicated was determined. Here, reliability not only reflects the degree of correlation but also the agreement between measurements (Daly and Bourke, 2008). Intraclass correlation coefficient (ICC) is an index that reflects both correlation and agreement between measurements. A two-way mixed-effect model selected using the ICC guidelines (Koo and Li, 2016), based on single rating, assessed the repeatability between the automated method and the ground truth. According to the ICC guidelines (Koo and Li, 2016), this specific ICC is most suitable for determining the consistency between two measurement methods. Interpretation of the ICC was as follows: <0.50, poor; between 0.50 and 0.75, fair; between 0.75 and 0.90 good; above 0.90, excellent.

Moreover, the computed axon morphometrics were tested for normality by the Shapiro-Wilks test, and group differences between the ground truth and automated segmentation were tested with the Wilcoxon signed rank test. To visually compare the agreement between the measurements from the automated method and the ground truth, we employed a Bland–Altman analysis with a significance level of 5%. All mean values are reported with their standard deviation.

**Figure 1:**
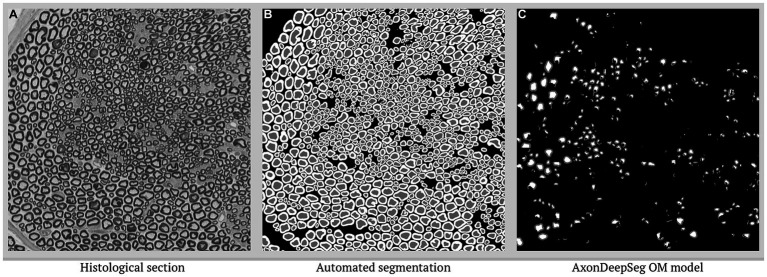
Segmentation comparison to AxonDeepSeg OM model. **(A)** Histological section of interest with **(B)** segmentation according to the presented algorithm, compared with **(C)** AxonDeepSeg OM model without any transfer learning.

For final validation of the presented algorithm, to determine reliability and repeatability of the automated method, the g-ratio, axon diameter and myelin thickness were calculated on three parallel histological sections of the same nerve fiber (Figure 1). Consequently, statistical testing was performed with a one-way ANOVA. All statistical analyses mentioned were performed in Python and R.

## 3 Results

**Figure 2:**
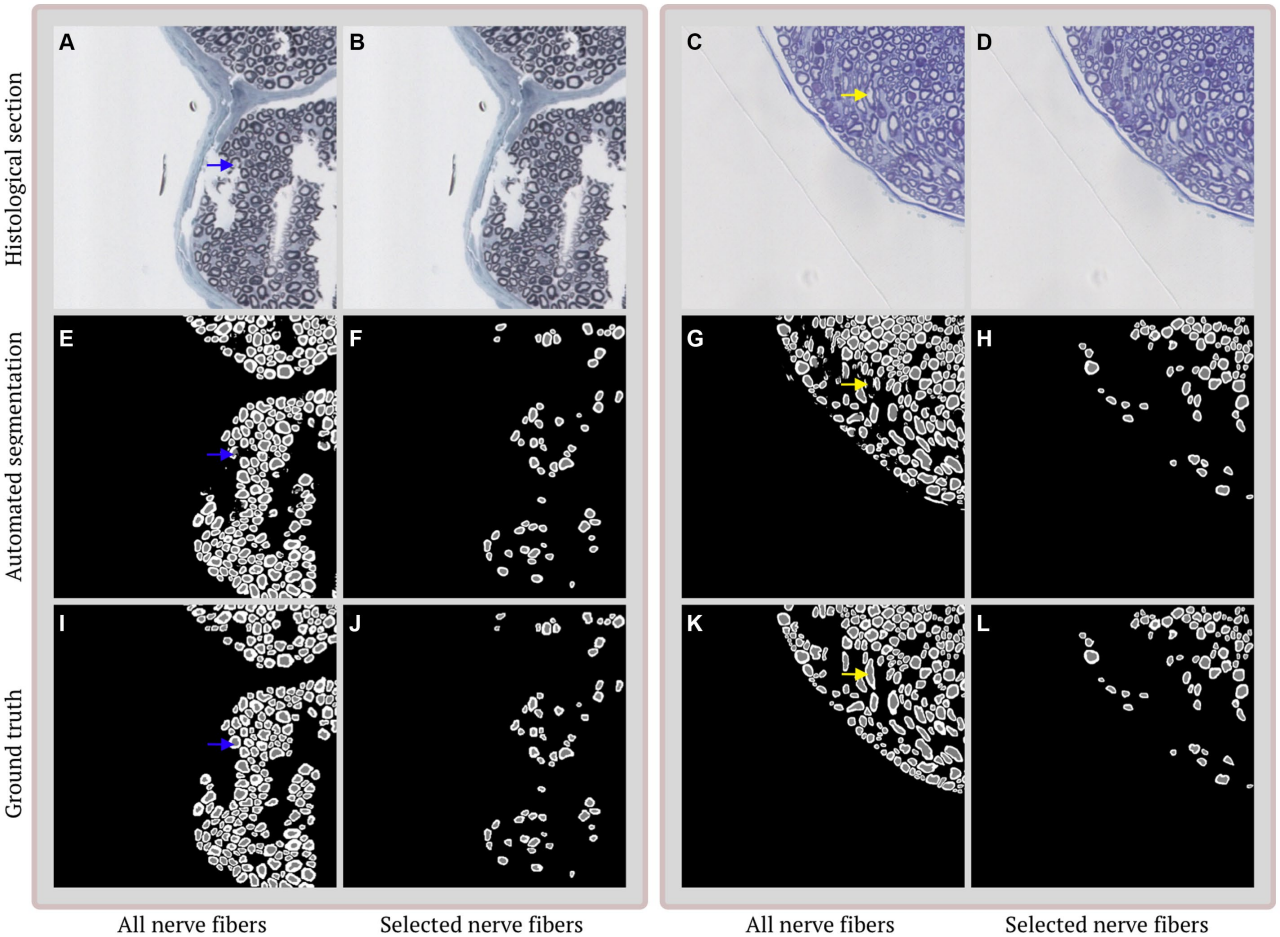
Examples of segmentation and selection performance on different histological sections. **(A,B)** Duplicate image of selected imperfect histological section stained with toluidine blue. **(C,D)** Duplicate image of selected histological section with multiple obliquely cut nerve fibers stained with 2%-p-phenylenediamine. **(E,G)** Automated segmentation of the histological section in **(A/C,B/D)**. The segmentations contain 3 classes: axon (gray), myelin (white) and background (black) and show a strong overall agreement. Discrepancies are mainly caused by incomplete (blue arrow) and obliquely (yellow arrow) cut nerve fibers. **(F,H)** Selection of nerve fibers that are suitable for morphological calculations. **(I,K)** Ground truth annotation corresponding to the histological section in **(A/C,B/D)**. **(J,L)** Selected ground truth annotations based on the selections in **(F,H)**.

Application of AxonDeepSeg (Zaimi et al., 2018) to our dataset resulted in under segmentation of the majority of the nerve bundles in our test set (Figure 1). Performance of our algorithm on the test set (Figure 2), prior to nerve fiber selection of OM, from ischiatic mouse nerves shows strong agreement with dice scores of 0.86 (axon) and 0.80 (myelin), and a pixel-wise accuracy of 0.98 (axon) and 0.94 (myelin). The density of nerve fibers does not seem to influence the ability of the algorithm to separate individual nerve fibers from each other. Following the selection of nerves that are suitable for g-ratio calculation, both dice scores increase (axon: 0.88, myelin: 0.84) as well as the pixel-wise accuracy (axon: 0.99, myelin: 0.98).

ICC for inter-device reliability of g-ratio (mean ICC 0.87, 95%CI [0.84, 0.89]) and myelin thickness (mean ICC 0.83, 95%CI [0.79, 0.85]) were good, and excellent for axon diameter (mean ICC 0.99, 95%CI [0.99, 0.99]), all features had a value of *p* < 0.001 indicating significance.

**Figure 3:**
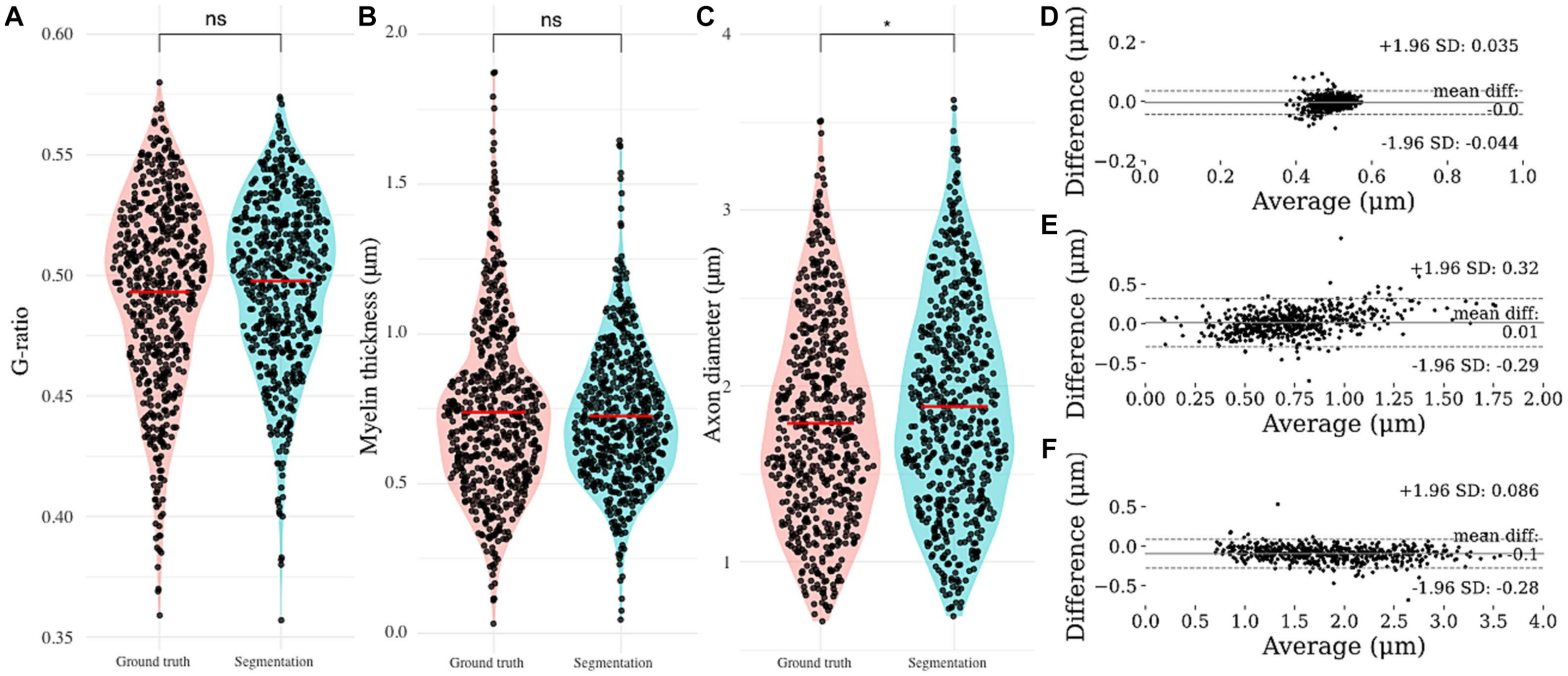
Automated nerve fiber segmentation morphometrics comparison. **(A)** Automatically obtained g-ratio, **(B)** myelin thickness and **(C)** axon diameter compared to the ground truth. Bland–Altman comparison of automated and ground truth **(D)** g-ratio, **(E)** myelin thickness and **(F)** axon diameter. Based on the Bland–Altman plot, g-ratio and axon diameter show excellent agreement, with myelin thickness giving an indication for a proportional bias.

Neither of the morphological parameters (axon diameter, myelin thickness and g-ratio) of the nerve fibers that were used for validation of the algorithm proved normally distributed on the Shapiro-Wilks test. The Wilcoxon signed-rank test showed no significant differences between the automated segmentation and ground truth regarding g-ratio (Figure 3A; *p* = 0.098) and myelin thickness (Figure 3B; 0.877), in contrast to axon diameter (Figure 3C; *p* < 0.01).

**Figure 4:**
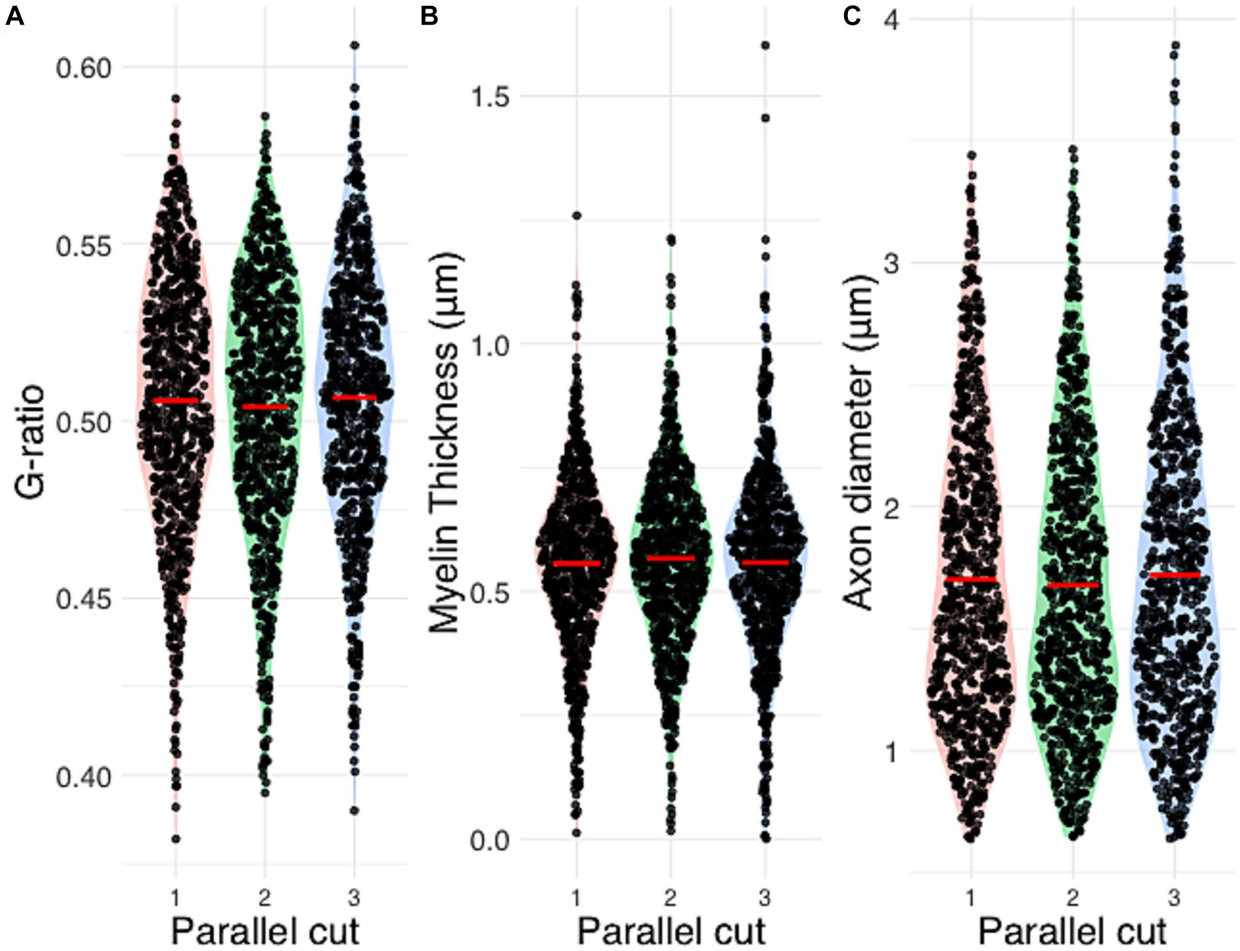
Automated segmentation morphometrics of three parallel histological sections. Inter-cut comparison of **(A)** g-ratio, **(B)** myelin thickness and **(C)** axon diameter. ANOVA *p*-values are reported as 0.34 (g-ratio), 0.50 (myelin thickness) and 0.39 (axon diameter).

Figure 4 presents the distributions of g-ratio, myelin thickness and axon diameter in the three parallel histological sections of the same nerve fiber. Although the algorithm detected a slightly deviating nerve fiber count (756 vs. 799 vs. 787), no statistical difference was shown on the morphological parameters. The ANOVA *p*-values are reported as 0.34 (g-ratio), 0.50 (myelin thickness) and 0.39 (axon diameter).

**Figure 5:**
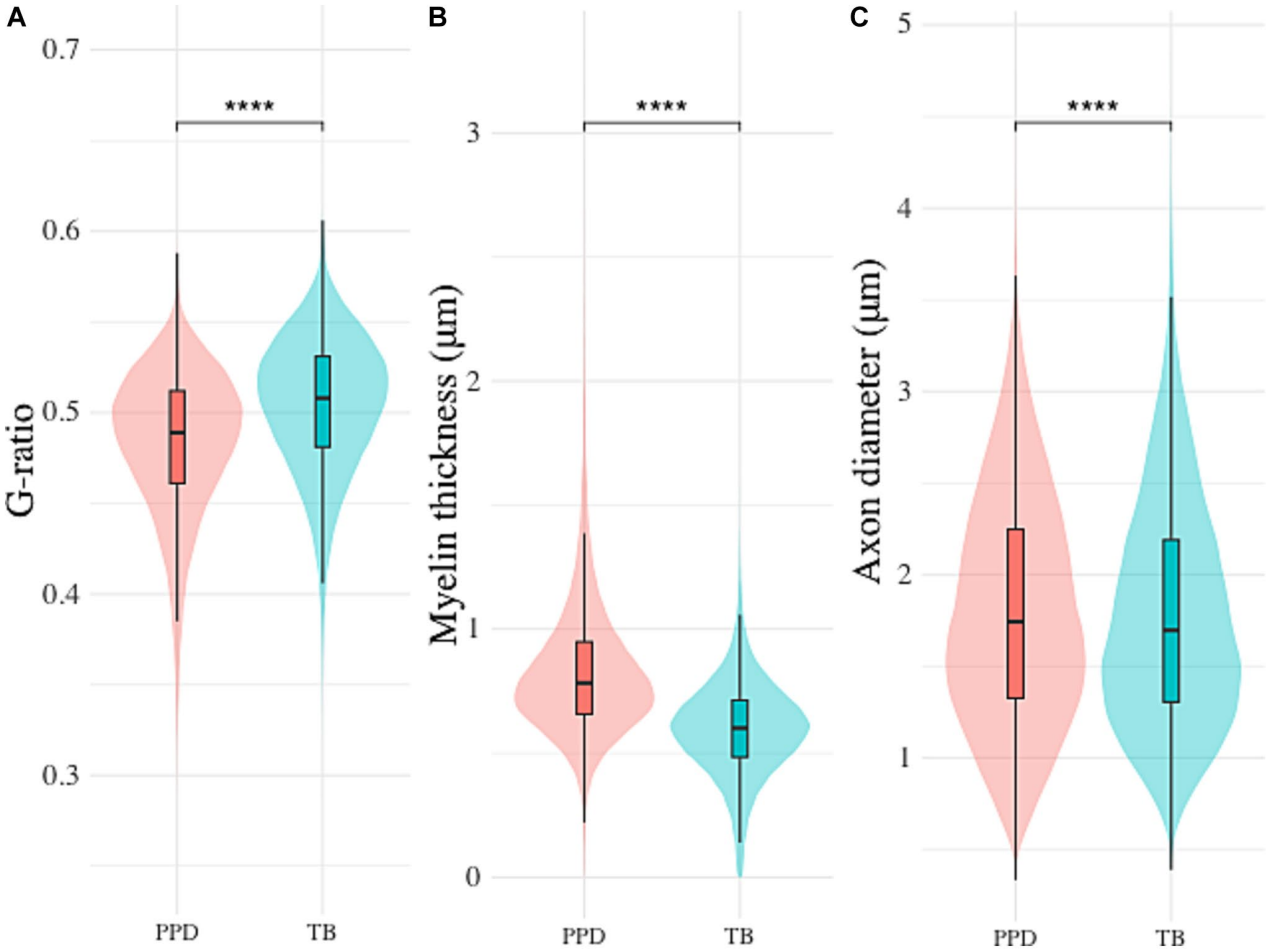
Differences in automated segmentation morphometrics between Toluidine blue (TB) and p-Phenylenediamine (PPD) staining. Inter-staining comparison of **(A)** g-ratio, **(B)** myelin thickness and **(C)** axon diameter (**** indicates *p* < 0.001).

In staining comparison, an average of 4914.0 (± 2370.1, range 458–8,403, PPD) and 5694.6 (± 5214.0, range 255–18,861, TB) nerve fibers were included in the morphometrical analysis per mouse. Mean g-ratio (PPD: 0.48 ± 0.04, TB: 0.50 ± 0.04), myelin thickness (PPD: 0.83 ± 0.28 μm, TTB: 0.60 ± 0.20 μm) and axon diameter (PPD: 1.80 ± 0.63 μm, TB: 1.78 ± 0.63 μm) are reported. None of the parameters proved normally distributed. By Wilcoxon signed-rank test, all morphological parameters were found to differ significantly between the two staining techniques (*p* < 0.001; Figure 5).

## 4 Discussion

The main objective of this study was to develop an automated pipeline for selection of nerve fibers that are relevant for g-ratio calculation based on quality parameters in optical microscopy. We provide strong evidence for automated calculations of the g-ratio as well as an overall segmentation performance that is similar to other methodologies that have been previously described (Zaimi et al., 2016, 2018; Deng et al., 2021; Kaiser et al., 2021). Collectively, the current study presents a methodology for g-ratio assessment, effectively revealing potential interpretational discrepancies between the two established staining protocols for OM.

In the current study we are able to reliably measure g-ratio and myelin thickness, but not axon diameter when compared to the ground truth. Although axon diameter is underlying for the g-ratio, the significant difference between the ground truth and automated segmentation does not appear to affect the g-ratio calculation. Possibly this is due to the way that the g-ratio is calculated, namely via AVF and MVF rather than the diameter of either axon or myelin. Moreover, demonstrating similar morphometrics on parallel histological sections of the same nerve fiber confirm the potential of the presented methodology. The architecture of our presented pipeline is similar to other methodologies that have been previously described (Zaimi et al., 2018; Janjic et al., 2019; Deng et al., 2021), with an overall segmentation performance that is also similar to what is presented in these methodologies. Of these, AxonDeepSeg provides the most elaborate framework (Zaimi et al., 2018), which has been adjusted to specific segmentation challenges with (Daeschler et al., 2022) and without (Wong et al., 2021) transfer learning in OM and EM, respectively. The segmentation metrics in our study prove similar to the strong performance of Daeschler et al. (2022). However, generalization of their model appeared to be challenging with our data, resulting in poor segmentation performance when applying their model to our data. Additionally, despite the strong segmentation performance presented in those papers, the automatically calculated g-ratio statistically deviated from its ground truth in both studies, in contrast to our study. These findings position our pipeline as a potential pre-selection methodology of large datasets, as a first round of quality control, prior to further evaluation on EM.

Given the complexities and variability of nerve fiber samples, a comprehensive and reliable measurement necessitates the inclusion of a large number of nerve fibers (Naito et al., 2017). Our presented pipeline showcased robustness and accuracy in measuring and analyzing g-ratio and myelin thickness in single parallel cuts from one animal, providing a solid foundation for this study. Moreover, our automated method enables eliminating the manual selection bias (Geuna et al., 2004) and ensuring more consistent results in g-ratio calculations. Selecting a representative and relevant number of nerve fibers increases the robustness of the analysis and implement standardization in a complex environment, wherein manual assessments are susceptible to bias (Figure 4). The pipeline has the ability to automatically select nerve fibers that are considered representative of the whole nerve for clinical evaluation. Consequently, differences in morphometric parameters were identified that could be due to the precise location of the tissue sectioning and the animal from which the sample was obtained. Additionally, the differences between TB and PPD staining underscore the need to carefully consider staining protocols in the morphometric analysis. We have shown that differences in staining methods significantly influence morphometric analysis (Figure 5). These differences in staining methods highlight the need for a careful choice of staining method. This specifically highlights the importance of carefully selecting staining and analysis methods for more accurate and reliable outcomes. Moving toward minimal errors and consistency, it is crucial to adopt automated and standardized approaches. Our findings highlight the need for future research to focus specifically on these variables, aiming to improve the accuracy of g-ratio measurements. This would add value for studies describing g-ratio effects due to neurodevelopment or pathological findings.

Despite its strengths, there are some limitations to our pipeline that should be addressed in future development. In case of large sample analysis, images have to be extracted from histological imaging software. To address this, automation could be implemented to enhance usability. Integrating the pipeline into Qupath, an open-source image analysis platform, could make it more widely accessible to researchers. Such integration could considerably enhance its accessibility and practical application. The ability of Qupath to handle large, multi-dimensional images from various sources combined with our efficient nerve selection and morphometrics calculation algorithm has the potential to streamline nerve fiber analysis, increasing the reproducibility and precision of results while minimizing manual intervention. Despite these potential advancements, the issue of generalizability that we have shown AxonDeepSeg to struggle with, might be applicable to our algorithm too, and thus warrants further research. Potential causes that limit generalizability are that our dataset is comprised of female mice only, which could have an effect on the g-ratio presentation (Paus and Toro, 2009). Furthermore, we have only included nerves of healthy animals into our dataset which expectedly results in reduced generalizability to pathological nerves sections. Further research would warrant including more diverse histological sections. Another aspect that was not included in the current study but would be valuable for future work is the assessment of inter-rater variability in g-ratio calculations, although this was not within the scope of our study given its focus on comparing different staining methods.

In conclusion, this study presents a new pipeline for automated g-ratio calculation based on OM, which has a strong performance with clinical benefit. Automation of g-ratio calculation can greatly reduce the time and effort required for manual measurements and increase the reproducibility and accuracy of results. Further development of the pipeline and integration into research software would improve usability. Additionally, careful interpretation across staining techniques is warranted.

## Data availability statement

The proposed pipeline and the dataset that have been used in development of the algorithm are available via GitHub (https://github.com/BartTh/g_ratio_selection) and Zenodo (https://doi.org/10.5281/zenodo.7642297).

## Ethics statement

The animal study was approved by the Cantonal Veterinary Office (animal permission number: ZH029/19) and follows the ARRIVE guidelines. The study was conducted in accordance with the local legislation and institutional requirements.

## Author contributions

BT: Data curation, Formal analysis, Investigation, Software, Validation, Writing – original draft, Writing – review & editing. LM: Investigation, Writing – review & editing. PS: Writing – review & editing, Data curation. HS: Data curation, Writing – review & editing. AS: Data curation, Writing – review & editing, Methodology. HR: Data curation, Methodology, Writing – review & editing, Conceptualization, Formal analysis, Investigation, Project–administration, Software, Validation, Writing – original draft, Visualization.

## Footnotes

^1^https://qupath.github.io
